# Family cohesion and adaptability reduces mobile phone addiction: the mediating and moderating roles of automatic thoughts and peer attachment

**DOI:** 10.3389/fpsyg.2023.1122943

**Published:** 2023-06-15

**Authors:** Shuai-Lei Lian, Xiao-Xuan Cao, Qing-Lu Xiao, Xiao-Wei Zhu, Chen Yang, Qing-Qi Liu

**Affiliations:** ^1^College of Education and Sports Science, Yangtze University, Jingzhou, China; ^2^Key Laboratory of Adolescent Cyberpsychology and Behavior (CCNU), Ministry of Education, Wuhan, China; ^3^Research Group Milestones of Early Cognitive Development, Max Planck Institute for Human Cognitive and Brain Sciences, Leipzig, Germany; ^4^College of Education for the Future, Beijing Normal University, Zhuhai, China

**Keywords:** family cohesion and adaptability, mobile phone addiction, automatic thoughts, peer attachment, adolescence

## Abstract

With the popularity of mobile Internet devices, the incidence of mobile phone addiction has been increasing, which has aroused the concern of all sectors of society. Due to the difficulty of eliminating the risk factors of mobile phone addiction, it’s significant for researchers to examine the function and underlying mechanisms of positive environmental factors in reducing individuals’ mobile phone addiction. Thus, the current study aimed to examine the relationship between family cohesion and adaptability and mobile phone addiction among university students and analyzed the mediating role of automatic thoughts as well as the moderating role of peer attachment in this link. The sample consisted of 958 Chinese university students. Participants completed self-report questionnaires assessing family cohesion and adaptability, mobile phone addiction, automatic thoughts, and peer attachment. PROCESS model 8 was significant (the total effect model (*F* (5, 952) = 19.64, *R*^2^ = 0.09, *p* < 0.001)). Results indicated that family cohesion and adaptability could not only negatively predict mobile phone addiction directly, but also indirectly through the mediating effect of automatic thoughts. Moreover, both the direct association between family cohesion and adaptability and mobile phone addiction as well as the indirect effect of automatic thoughts were moderated by peer attachment. Findings emphasized the beneficial role of peer attachment on the effect of family cohesion and adaptability on automatic thoughts and mobile phone addiction.

## 1. Introduction

With the rapid development of digital technology and the widespread use of mobile Internet devices, mobile phones have been ubiquitously used around the world and become an essential part of modern daily life (Hu et al., 2020; Liu et al., 2022). According to the report by the China Internet Network Information Center, the number of people who reported accessing the Internet through mobile phones has reached 1065 million, and the population of mobile phone users has grown rapidly (China Internet Network Information Center, 2023). Unfortunately, this universal and extensive use of mobile phones has coincided with the emergence of problematic behaviors such as mobile phone addiction (Geng et al., 2021). Mobile phone addiction refers to excessive or uncontrolled mobile phone use in daily life, which can exert an adverse influence on both physiological and psychological health (Scott et al., 2020). Growing evidence has revealed that mobile phone addiction is closely related to a variety of problems such as social isolation (Al-Kandari and Al-Sejari, 2020), poor sleep quality (Liu et al., 2017), and psychological distress (Lian et al., 2020). At the same time, this non-substance addiction is especially prominent among adolescents and young adults, so it is necessary to examine the addiction of mobile phone addiction and its mechanism of action among college students in depth (Diotaiuti et al., 2022a,b).

Previous researchers revealed plenty of risk factors for mobile phone addiction, such as peer victimization (Liu et al., 2020a), parental rejection (Chen Y. et al., 2021), and loneliness (Liu et al., 2021). As more and more risk factors of mobile phone addiction have been found, researchers have examined the protective factors of mobile phone addiction from the perspective of reducing risk factors (Liu et al., 2019). However, due to the multiformity and complexity of risk factors, it was difficult for us to eliminate the risk factors of mobile phone addiction and reduce the possibility of individuals being addicted to mobile phones. Therefore, an increasing number of people suggest that it is better to tell them what to do rather than telling them not to do it. Furthermore, researchers have increasingly emphasized the importance of examining the role and underlying mechanisms of positive environmental factors, such as creating supportive family or peer interaction environments, in reducing mobile phone addiction (Liu et al., 2020a; Zhang R. et al., 2021). It provides a new perspective on intervention research, that is, guiding individuals, families, or schools to build a positive development environment that can promote rational mobile phone use and reduce mobile phone addiction. Building on this perspective, the present study attempts to explore the association between positive family environmental factors (family cohesion and adaptability) and mobile phone addiction as well as the underlying mechanisms that could effectively reduce the risk of mobile phone addiction.

### 1.1. Family cohesion and adaptability and mobile phone addiction

Ralph Waldo Emerson, a famous ideologist and philosopher in the nineteenth century, wrote that *The family is the father’s kingdom, the mother’s world, and the children’s paradise*, expressing high praise for a family and emphasizing the important role of family for every family member. Similarly, plenty of ancient Chinese literati and artists also created countless immortal works to convey the thoughts of a family and their yearning for harmonious and beatific family life, such as *jollification of reuniting family, such kindness of warm sun, cannot be repaid by grass*. All of these manifested that the positive meaning of family to individuals’ development has cross-cultural universality. In addition, previous studies have revealed the positive effects of family factors on individuals’ emotional and behavioral adaptation, such as family support (Wills et al., 1992), parent–child interaction (Zimmer-Gembeck, 2011), and family socioeconomic status (Sarsour et al., 2011). The positive role of family cohesion and adaptability in individuals’ psychological and behavioral development has also drawn the attention of many researchers (Ye et al., 2021; Li M. et al., 2021).

Family cohesion and adaptability have been considered as a comprehensive measure of the degree of family functioning (Bakken and Romig, 1989). Cohesion refers to “the emotional bond between members and the degree of autonomy experienced by a person in the family system.” Adaptability refers to “the ability of the marriage or family system to change its power structure, role relationships, and relationship rules based on context and development pressure.” Prior studies have shown that family cohesion and adaptability might be protective factors in mitigating both the risk of indulging in the Internet and the negative consequences associated with Internet addiction (Yen et al., 2007; Liu et al., 2020b).

First, family cohesion and adaptability could reduce the risk of individuals being entangled in emotional problems and thereby reduce the possibility of individuals indulging in mobile phones. Previous studies have demonstrated that emotional problems such as loneliness and anxiety are important triggers for individuals to indulge in mobile phones (Liu et al., 2021; Li J. et al., 2021). Family cohesion and adaptability could provide emotional communication, psychological comfort and support to help family members overcome the emotional difficulties they encounter (Arató et al., 2021). The love and warmth offered by the family would reduce the possibility of loneliness and other emotional problems, as well as the potential adverse consequences associated with emotional problems (Lei and Kantor, 2021). Therefore, families with higher cohesion and adaptability could satisfy individuals’ emotional needs and reduce the risk of individuals being addicted to mobile phones.

Besides, family cohesion and adaptability could help individuals to shape positive personality traits and enhance their social adaptability (Yan et al., 2014). Positive personality traits and good social adaptability may also help individuals reduce the possibility of escaping from reality due to frustration and indulging in the virtual world. According to the ACE model (Young, 1998), escapism is an important motivation for mobile phone addiction. A previous study has also stated that individuals who grow up in families with poor cohesion and adaptability were more likely to escape from reality when encountering setbacks, and then show a higher tendency to indulge in mobile phones (Arató et al., 2021). Whereas individuals with stronger adaptability will seek assistance from others rather than escaping from reality or getting lost in the mobile phones when faced with setbacks and difficulties (Lanfranchi and Vianello, 2012).

Moreover, family cohesion and adaptability could act as a security base to reduce individuals’ behavioral problems, such as mobile phone addiction. Previous studies have stated that family cohesion and adaptability could promote individuals’ emotional security, which helps individuals to form positive behavior habits and effective stress-coping styles (Bakken and Romig, 1989; Sela et al., 2019). In addition, emotional safety is significantly negatively correlated with mobile phone addiction (Hong et al., 2020). According to Olson’s Circumplex Model (Schweitzer, 1992), strong emotional bonds within the family can help individuals maintain stable physical and mental health, and reduce their internalizing or externalizing problems. Conversely, dysfunctional and conflict-ribben families could increase the social adjustment problems of adolescents, which contributes to engagement in mobile phone addiction (Liu et al., 2020b). Above all, we supposed that family cohesion and adaptability would be negatively associated with mobile phone addiction (Hypothesis 1).

### 1.2. Automatic thoughts as the mediator

Automatic thoughts refer to automatic, recurring negative thinking patterns caused by cognitive schema or core belief (Beck, 1967). They have been identified as crucial risk factors for maladaptive coping strategies and problematic behaviors (Hjemdal et al., 2013; Hou et al., 2020; Nie et al., 2021). Individuals with higher automatic thoughts are more likely to develop negative and self-defeating thoughts that are often activated by negative life experiences (Yapan et al., 2020). Once activated, automatic processes run to fulfillment and are difficult to alter or suppress (Irfan and Zulkefly, 2021). These intrusive thoughts can induce emotional problems, such as depression and anxiety. To relieve such negative emotions, individuals may turn to the virtual world (e.g., mobile phones). Over time, they become more and more addicted to mobile phones and find it difficult to extricate themselves (Liu et al., 2021). Prior studies have shown that mobile phones have been widely used as a tool to pass time and eliminate negative emotions (Khang et al., 2013). Therefore, the higher the frequency of automatic thoughts, the easier it is to fall into negative emotional states such as anxiety and depression. These negative emotions make it more likely to use mobile phones to escape from the real world, thus inducing mobile phone addiction. Besides, negative automatic thoughts are closely correlated with individuals’ way of mindsets and interpretations of situations, which in turn, lead to maladaptive coping strategies such as escape and mobile phone addiction. Previous studies have demonstrated that automatic thoughts play a key role in escaping from reality and indulging in the Internet (Geng et al., 2009; Nie et al., 2021). Specifically, individuals with high automatic thoughts are more likely to create automatic and passive thoughts when they encounter frustration, which may prompt their escapist behaviors and further induce mobile phone addiction. Moreover, it has been confirmed that individuals with higher automatic thoughts are more prone to Internet addiction (Geng et al., 2009). As another manifestation of Internet addiction in the mobile Internet era (Billieux et al., 2015), mobile phone addiction may also be a potential adverse consequence of automatic thoughts. Therefore, this study speculated that automatic thoughts may increase the risk of individuals being addicted to mobile phones.

Researchers have also indicated that automatic thoughts are closely correlated with the family environment (Kazdin, 1990). According to the ecological system theory, family is the primary place for individuals’ socialization, which relates to the formation of their cognitive styles, behavior habits, and values (Swartz and Martin, 1997). Empirical studies have shown that individuals from families with conflict-indifferent relationships report more negative automatic thoughts than those from harmonious families (Burgess and Haaga, 1994). That is, family cohesion and adaptability may reduce individuals’ automatic thoughts. Additionally, there is evidence that automatic thoughts play an intermediary role in the association between family environments and problem behaviors (Irfan and Zulkefly, 2021). Therefore, it was hypothesized that automatic thoughts may mediate the effect of family cohesion and adaptability on mobile phone addiction (Hypothesis 2).

### 1.3. Peer attachment as the moderator

Peer attachment refers to an enduring and close affectionate bond between individuals and their peers, which can provide warmth and support to each other (Bowlby, 1969; Li et al., 2022). As a social support resource, it has protective and beneficial effects on negative thinking styles and behavior adaptation (Armsden and Greenberg, 1987; Saija et al., 2022).

When facing conflicts and the pernicious impact of the family, individuals with high-quality attachment can constantly adjust their cognition and develop a positive cognition model which could reduce the possibility of being involved in negative automatic thoughts (Pace et al., 2011; Hou et al., 2020). In addition, they will seek assistance from peers and acquire more attention and comfort when facing negative emotions brought by undesirable families (Lei and Wu, 2009; Chen and Feng, 2013). Therefore, they may employ more valid and effective emotional coping strategies to regulate negative emotions and reduce the adverse effects of detrimental emotions, and ultimately reduce the risk of falling into externalizing problems such as indulging in mobile phones.

According to the development environment theory (Yarrow and Zaslow, 1981), individuals’ multiple environments do not independently relate to their development, but jointly determine the developmental outcomes through some interactions. Individuals who enter the university gradually move away from the close-knit family environment, and begin to interact with peers. Previous studies have shown that peer attachment can weaken the adverse effects of parental marital conflict and childhood abuse (Armsden and Greenberg, 1987; Yang et al., 2016). Therefore, peer attachment, as a positive environmental factor, can interact with the family environment to influence individuals’ psychological and behavioral development. The protection-protection factor model indicated that one protective factor can amplify or enhance the beneficial effects of another factor (Brook et al., 1986). Previous studies have demonstrated that family cohesion and adaptability and peer attachment were two important and effective protective factors that could reduce the cognitive and behavioral adaptation problems (Ko, 2009; Qing et al., 2018; Badenes-Ribera et al., 2019; Sela et al., 2019). Therefore, high-quality peer relationships may enhance the beneficial effects of high family cohesion and adaptability on individuals’ development. Empirical studies have observed that secure peer attachment could improve individuals’ emotional responses and thinking modes, which could enhance the positive impact of a satisfactory family environment on their thinking patterns (Li et al., 2017). Prior studies have also shown that high-quality peer attachment relationships could develop and reinforce individuals’ positive coping styles and thinking models, and this beneficial effect was greater in an intimate family environment (Badenes-Ribera et al., 2019). In addition, intimate families provide parental emotional warmth and support, coupled with high-quality peer attachment relationships, which could greatly increase individuals’ social adaptability and reduce the occurrence of problematic behaviors, such as mobile phone addiction (Qing et al., 2018). Prior studies have also indicated that parental emotional warmth is positively correlated with peer attachment, and they are both negatively associated with mobile phone addiction (Gao et al., 2022). Therefore, this study hypothesized that peer attachment could enhance the positive effects of family cohesion and adaptability, and further mitigate the risk of automatic thoughts on mobile phone addiction (Hypothesis 3).

### 1.4. The present study

Considering the prevalence and perniciousness of mobile phone addiction, and the protective role of family functions on individuals’ behavioral adaptation, it is imperative to examine the relationship between family cohesion and adaptability and mobile phone addiction. The current study attempts to examine the mediating effect of automatic thoughts and the moderating role of peer attachment in the association between family cohesion and adaptability and mobile phone addiction. This study provides a new perspective on the protective mechanisms of mobile phone addiction. The findings of this study would offer valuable suggestions for parents on how to cultivate intimate and harmonious family relationships, as well as guide individuals in developing high-quality peer attachment, thereby reducing the risk of mobile phone addiction.

## 2. Method

### 2.1. Participants and procedure

Convenience sampling was employed to recruit 958 students (59.3% female) from two universities located in the center of China (Wuhan and Jingzhou). Students who possessed smartphones and used them for both personal activities and professional studies were invited to participate in the study. All of the participants were informed of the study’s requirements by acquainting standard instructions carefully and were encouraged to answer truthfully and independently within 30 min in their classroom. The mean age of the participants was 19.90 years old (*SD* = 1.22). Three hundred and fifteen (32.9%) of them were freshmen; three hundred and forty-two (35.7%) of them were sophomores; and three hundred and one (31.4%) of them were juniors. The study obtained both Ethics Committee approval prior to conducting the study and informed consent from each student.

### 2.2. Measurements

#### 2.2.1. Family cohesion and adaptability

Family cohesion and adaptability were assessed by the Chinese version of the Family Adaptability and Cohesion Scale (Olson et al., 1985) and had shown good reliability and validity among Chinese college students (Ye et al., 2021). This scale is mainly applied to assess two aspects of family function including cohesion (e.g., emotional connection between family members) and adaptability (e.g., family system’s capabilities to overcome difficulties). Examples of items are “Family members are familiar with each other’s close friends.” The scale consists of 30 items (16 items for family cohesion and 14 items for family adaptability). Participants respond to the 30 items on a Likert-type scale ranging from 1 (never) to 5 (always). All scores are added together and averaged, higher scores represent more stable and harmonious family relationships. In the current study, the items demonstrated high reliability (Cronbach’s *α* = 0.91).

#### 2.2.2. Mobile phone addiction

Mobile phone addiction was measured by the Mobile Phone Addiction Index (MPAI; Leung, 2008). This scale consists of four dimensions including avoidance, inefficiency, out of control, and withdrawal (e.g., “You spend a lot of time on your mobile phone and are unable to control yourself”). Participants respond to the 17 items on a Likert-type scale ranging from 1 (never) to 5 (always). All scores are added together and averaged, higher scores represent severe mobile phone addiction. In the current study, the items demonstrated high reliability (Cronbach’s *α* = 0.85).

#### 2.2.3. Automatic thoughts

Automatic thoughts were assessed by the Automatic Thought Questionnaire (Hollon and Kendall, 1980) and had shown good reliability and validity among Chinese students (Yao et al., 2003). This scale consists of four dimensions including maladjustment and eagerness for change, negative self-concepts and expectations, lack of self-confidence, and helplessness (e.g., “I am very dissatisfied with myself”). Participants respond to the 30 items on a Likert-type scale ranging from 1 (did not emerge this thought at all) to 5 (continue to occur frequently). All scores are added together and averaged, higher scores reflect a higher tendency to automatically think of the negative life experience. In the current study, the items demonstrated high reliability (Cronbach’s *α* = 0.97).

#### 2.2.4. Peer attachment

Peer attachment was assessed by a shortened version of the Peer Attachment Inventory (Armsden and Greenberg, 1987). The reliability and validity of the Chinese version of this questionnaire have been tested and verified (Chen X. et al., 2021). The scale has 12 items scored on a 5-point Likert scale (1 = definitely not to, 5 = absolutely yes). Examples of items used are “If I want to tell my friends something, they hear me carefully.” All scores are added together and averaged, higher scores indicate more experience of companionship, love, security, and acceptance from friends. In the current study, the items demonstrated acceptable reliability (Cronbach’s *α* = 0.73).

#### 2.2.5. Control variables

Gender, age, grade, and years of owning a mobile phone were included as control variables in the current study, as prior researchers found that they were closely related to the main variables in this current study (Pavia et al., 2016; Liu et al., 2021; Zhang Y. et al., 2021).

### 2.3. Statistical analyses

Firstly, we conducted descriptive statistics to examine the means and standard deviations for gender, age, grade, years of owning a mobile phone, family cohesion and adaptability, mobile phone addiction, automatic thoughts, and peer attachment. We employed Pearson correlations to test the bivariate associations for all observed variables. Secondly, the SPSS macro PROCESS (model 8) was used to test the proposed moderated mediation model which was suggested by Hayes (2013) and has been widely used to test mediating and moderating models by several researchers (Liu et al., 2017, 2020a, 2021). Thirdly, simple slopes analyses were used to decompose the significant interaction effects in this study.

## 3. Results

### 3.1. Preliminary analyses

The observed variables’ means, standard deviations, and correlations are displayed in Table 1. As hypothesized, family cohesion and adaptability were negatively associated with automatic thoughts (*r* = −0.28, *p* < 0.01) and mobile phone addiction (*r* = −0.24, *p* < 0.01). Automatic thoughts were positively associated with mobile phone addiction (*r* = 0.38, *p* < 0.01). Peer attachment was positively associated with family cohesion and adaptability (*r* = 0.19, *p* < 0.01) while negatively associated with automatic thoughts (*r* = −0.08, *p* < 0.01) and mobile phone addiction (*r* = −0.07, *p* < 0.01).

**Table 1:** Descriptive statistics and interrelations among all of the observed variables.

Variables	*M*	SD	1	2	3	4	5	6
1. Age	19.90	1.22	1					
2. Years of owning a mobile phone	5.93	2.51	0.14^**^	1				
3. Family cohesion and adaptability	3.14	0.53	−0.01	−0.01	1			
4. Automatic thoughts	2.11	0.81	−0.02	0.07^*^	−0.28^**^	1		
5. Peer attachment	3.07	0.49	−0.04	−0.006	0.19^**^	−0.08^*^	1	
6. Mobile phone addiction	2.60	0.59	−0.05	0.11^**^	−0.24^**^	0.38^**^	−0.07^*^	1

*N* = 970. **p < 0.01, *p < 0.05.

### 3.2. Testing for the proposed moderated mediation model

Hayes’s (2013) SPSS macro PROCESS was adopted to examine the proposed moderated mediation model. Table 2 presented the main results.

**Table 2:** Regression results for the conditional direct and indirect effects.

*Model: Total effect model*									
*R*	*R* ^2^	*F*	*df_1_*	*df_2_*	*p*	B	*SE*	*t*	*p*
0.30	0.09	19.64	5	952	< 0.001				
Constant						3.69***	0.45	8.23	<0.001
Gender						0.17***	0.04	4.31	<0.001
Age						−0.04	0.02	−1.56	>0.05
Grade						0.02	0.03	0.51	>0.05
Years						0.03***	0.01	3.35	<0.001
Family cohesion and adaptability						−0.26***	0.03	−8.32	<0.001
**Model: Mediator variable model**									
*R*	*R* ^2^	*F*	*df_1_*	*df_2_*	*p*	B	*SE*	*t*	*p*
0.33	0.11	24.30	7	950	< 0.001				
Constant						2.38***	0.59	4.02	<0.001
Gender						−0.21***	0.05	−3.88	<0.001
Age						0.002	0.03	0.59	>0.05
Grade						−0.05	0.05	−1.09	>0.05
Years						0.02*	0.01	2.18	<0.05
Family cohesion and adaptability						−0.38***	0.05	−8.29	<0.001
Peer attachment						−0.01	0.06	−0.24	>0.05
Family cohesion and adaptability × peer attachment						−0.20**	0.06	−3.30	<0.01
**Model: Dependent variable model**									
*R*	*R* ^2^	*F*	*df_1_*	*df_2_*	*p*	B	*SE*	*t*	*p*
0.46	0.21	30.06	8	949	<0.001				
Constant						2.20***	0.39	5.62	<0.001
Gender						0.22***	0.04	6.07	<0.001
Age						−0.03	0.02	−1.56	>0.05
Grade						0.03	0.03	0.89	>0.05
Years						0.02*	0.007	2.57	<0.05
Automatic thoughts						0.26***	0.24	10.64	<0.001
Family cohesion and adaptability						−0.11***	0.31	−3.68	<0.001
Peer attachment						0.003	0.04	0.07	>0.05
Family cohesion and adaptability × peer attachment						−0.13*	0.06	−2.36	<0.05
*Conditional direct effect analysis at values of peer attachment* (*M ±* SD)						B	Boot *SE*	Boot LLCI	Boot ULCI
*M* − 1SD (2.58)						−0.05	0.04	−0.13	0.04
*M* (3.07)						−0.11	0.03	−0.17	−0.05
*M* + 1SD (3.56)						−0.18	0.04	−0.26	−0.10
*Conditional indirect effect analysis at values of peer attachment* (*M ±* SD)						B	Boot *SE*	Boot LLCI	Boot ULCI
*M* − 1SD (2.58)						−0.07	0.02	−0.11	−0.04
*M* (3.07)						−0.10	0.01	−0.13	−0.07
*M* + 1SD (3.56)						−0.12	0.02	−0.16	−0.10

*N* = 970. Unstandardized regression coefficients are reported. Bootstrap sample size = 5,000. LL, low limit, CI, confidence interval, UL, upper limit. ^*^p < 0.05. ^**^p < 0.01. ^***^p < 0.001.

As expected, the total effect model (*F* (5, 952) = 19.64, *R*^2^ = 0.09, *p* < 0.001), the mediator variable model (*F* (7, 950) = 24.30, *R*^2^ = 0.11, *p* < 0.001) and dependent variable model (*F* (8, 949) = 30.06, *R*^2^ = 0.21, *p* < 0.001) were all significant after controlling gender, age, grade and years of owning a mobile phone. Specifically, family cohesion and adaptability were negatively associated with automatic thought (*B* = −0.38, *p* < 0.001) and mobile phone addiction (*B* = −0.11, *p* < 0.001). Automatic thoughts were positively associated with mobile phone addiction (*B* = 0.26, *p* < 0.001). Furthermore, we employed the Sobel test to examine the significance of the indirect effect of family cohesion and adaptability on mobile phone addiction via automatic thought. The results indicated that automatic thoughts significantly mediated the relationship between family cohesion and adaptability and mobile phone addiction (*z* = 10.91, *p* < 0.001). These results provided compelling evidence that family cohesion and adaptability were associated with decreasing mobile phone addiction and this relation was mediated by automatic thoughts. Thus, Hypotheses 1 and 2 were supported.

In order to examine Hypothesis 3, Hayes’s (2013) PROCESS macro (Model 8) was adopted to analyze the two interaction effects. There were a significant family cohesion and adaptability × peer attachment interaction effect on automatic thoughts (*B* = −0.20, *p* < 0.01) in the mediator variable model and a significant family cohesion and adaptability × peer attachment interaction effect on mobile phone addiction (*B* = −0.13, *p* < 0.05) in the dependent variable model. These findings indicated that both the association between family cohesion and adaptability and mobile phone addiction and the association between family cohesion and adaptability and automatic thoughts were moderated by peer attachment.

**Figure 1:**
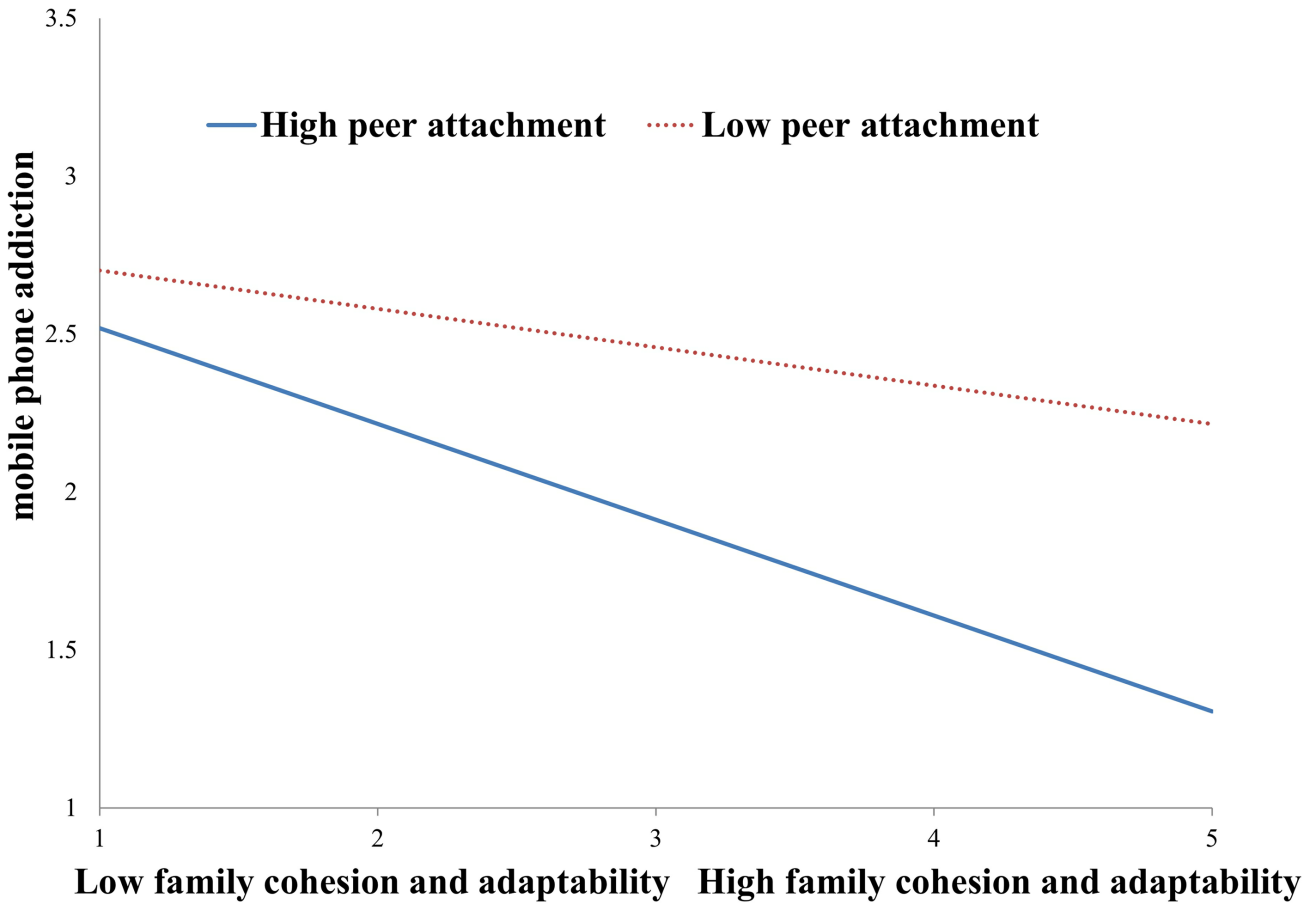
Peer attachment moderated the relationship between family cohesion and adaptability and mobile phone addiction.

**Figure 2:**
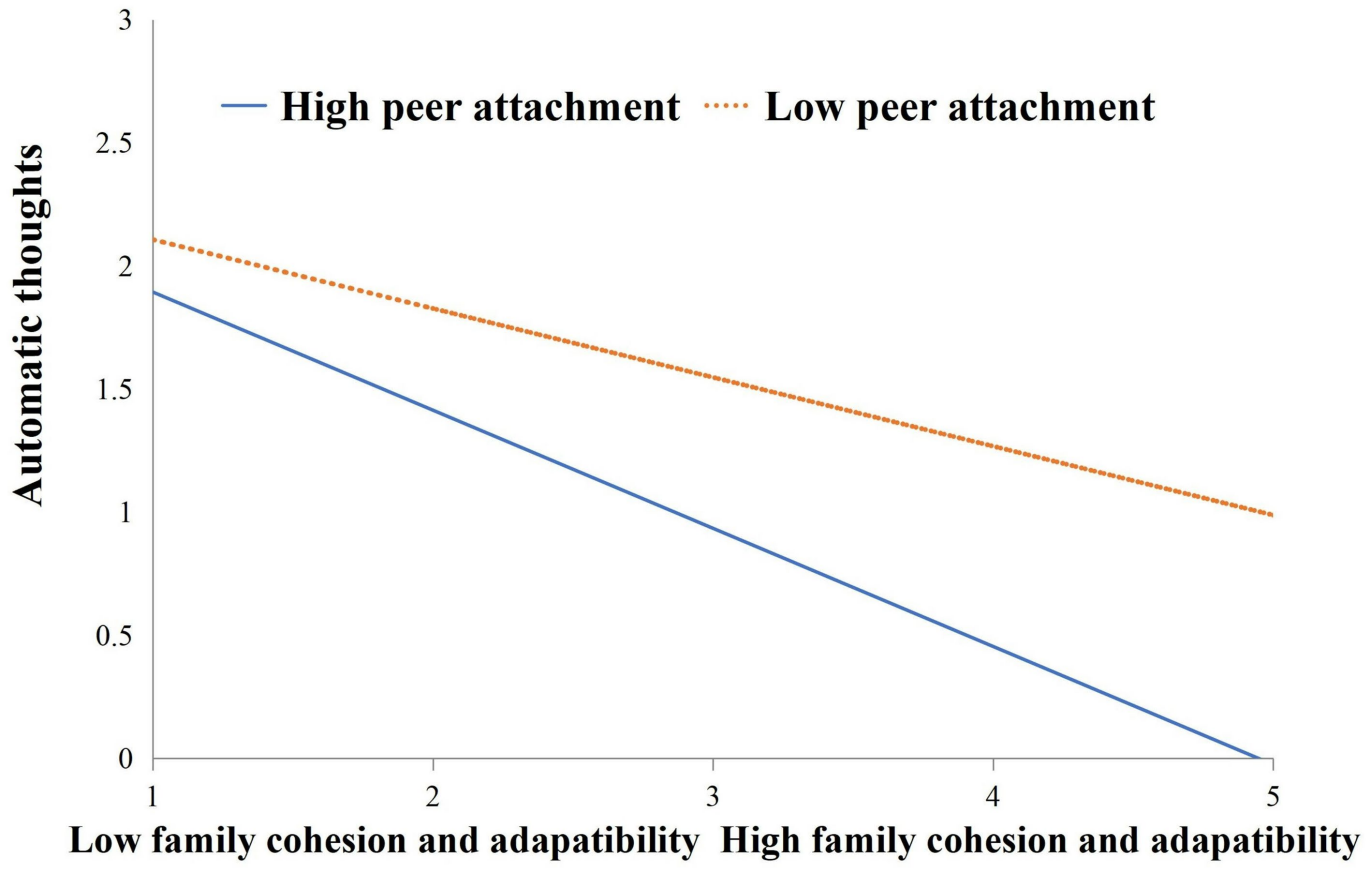
Peer attachment moderated the relationship between family cohesion and adaptability and automatic thoughts.

Additionally, we conducted simple slope analyses to illustrate these significant interactions and explore whether slopes for the high-peer attachment group (1 *SD* above the mean) were different from slopes for the low-peer attachment group (1 *SD* below the mean) in the two models. The results were plotted in Figures 1, 2. As shown in Figure 1, the negative effect of family cohesion and adaptability on automatic thoughts was stronger for students with higher peer attachment (*B* = −0.30, *t* = −7.53, *p* < 0.001) than for those with lower peer attachment (*B* = −0.12, *t* = −2.71, *p* < 0.01). Similarly, as shown in Figure 2, the negative effect of family cohesion and adaptability on mobile phone addiction was stronger for students with higher peer attachment (*B* = −0.48, *t* = −10.39, *p* < 0.001) than for those with lower peer attachment (*B* = −0.28, *t* = −4.48, *p* < 0.001). In other words, family cohesion and adaptability interacted with peer attachment, so students with low levels of peer attachment had fairly similar automatic thoughts and mobile phone addiction across high and low levels of family cohesion and adaptability. However, students with higher levels of peer attachment reported lower levels of automatic thoughts and mobile phone addiction when they had higher family cohesion and adaptability. Likewise, students with lower levels of peer attachment reported higher levels of mobile phone addiction and automatic thoughts.

Furthermore, the two conditional analyses showed that no matter what levels of peer attachment are, all of the direct and indirect effects were negatively and significantly different from zero. Namely, both the direct effect of family cohesion and adaptability on mobile phone addiction and the indirect effect of automatic thoughts in this link were stronger for students with higher peer attachment.

## 4. Discussion

The present study examined the association between family cohesion and adaptability and mobile phone addiction, and the mediating role of automatic thoughts in this link. In addition, peer attachment was tested as a moderator in this mediation process. The results showed that family cohesion and adaptability were negatively associated with mobile phone addiction. Hypothesis 1 was supported. The moderated mediation analysis revealed that automatic thoughts mediated the association between family cohesion and adaptability and mobile phone addiction. Hypothesis 2 was supported. Moreover, the direct and indirect effects of family cohesion and adaptability on mobile phone addiction could be moderated by peer attachment. Specifically, individuals with higher levels of peer attachment could successfully enhance the positive effects of family cohesion and adaptability on automatic thoughts and mobile phone addiction. Hypothesis 3 was supported. By shedding light on both how and when family cohesion and adaptability are negatively associated with mobile phone addiction, this study could deepen our understanding of the role of family function in mobile phone addiction. To our knowledge, this is the first study that tested both the mediation and moderation mechanisms underlying the link between family cohesion and adaptability and mobile phone addiction. Moreover, the present study may inform the design of intervention programs aimed at reducing mobile phone addiction among college students.

Consistent with previous studies, individuals who grow up in intimate families and feel more emotional warmth can develop secure attachments and regard family members as faithful friends, thereby reducing the likelihood of mobile phone addiction (Liu et al., 2020b). On the contrary, individuals who live in families with low levels of cohesion and adaptability for a long time may experience more anxiety and loneliness (Gao et al., 2022). They may fail to establish intimate relationships with others and fear being abandoned constantly, and thus treat mobile phones as attachment objects and be addicted to mobile phones ultimately (Parent et al., 2021). This conclusion coincided with the pathway model of mobile phone addiction (Billieux et al., 2015), such that mobile phone addiction was caused by cognitive dissonance or the need for relationship maintenance due to insecure attachment. Besides, individuals living in high-intimacy families tend to be harmonious, humorous, and supportive, experience more fun and self-worth, have a stronger sense of control and life meaning (Cheng et al., 2019). They would have a clear sense of purpose and tend to live in accordance with their values or goals, thus being less addicted to mobile phones (Zhang et al., 2019). Conversely, individuals who grow up in isolated and dysfunctional families have a low sense of meaning in life, have no clear direction for life, and often experience negative emotions such as loneliness and anxiety. Therefore, they seek entertainment and the meaning of life and alleviate negative emotions by escaping from reality in the virtual mobile world, which increases the risk of mobile phone addiction (Chen Y. et al., 2021).

Besides, our findings were consistent with previous studies, showing that automatic thoughts acted as a mediating role that linked family cohesion and adaptability to mobile phone addiction (Irfan and Zulkefly, 2021; Nie et al., 2021). This finding indicated that individuals living in families with low cohesion and adaptability tended to develop negative, automatic, irrational beliefs, and negative emotions, which eventually led to problematic behaviors such as mobile phone addiction. This conclusion coincided with Beck’s cognitive theory of mood disorders (Beck, 1976), emphasizing the important role of thoughts and beliefs in behavior and dysfunction. Our mediation model of automatic thoughts was also consistent with previous studies indicating that automatic thoughts are an important proximal factor, through which an undesirable family environment was linked to physical and mental development (Kazdin, 1990; Hou et al., 2020). Individuals from families with more parental conflicts and undesirable parent–child relationships were more likely to develop characteristics such as low self-evaluation and low self-esteem, which were reflected through individuals’ negative automatic thoughts (Irfan and Zulkefly, 2020). These negative cognitive styles would enhance individuals’ negative internal working model, it may motivate individuals to employ negative coping skills such as escaping from reality and indulging in virtual mobile phones when they make excessive inferences about negative stimuli (Hjemdal et al., 2013; Irfan and Zulkefly, 2021).

Furthermore, a most important finding in the present study was the individual differences in the effects of family cohesion and adaptability on automatic thoughts and mobile phone addiction. Specifically, both the direct effect that family cohesion and adaptability itself exerted on mobile phone addiction and the indirect effect via automatic thoughts were moderated and enhanced by peer attachment, with these effects being stronger for individuals with a higher level of peer attachment. Individuals can feel more secure and being loved when they possess both high-cohesion and adaptability families and high-quality peer relationships, which could alleviate negative thoughts and the risk of mobile phone addiction by reducing negative emotional experiences (Liu et al., 2020a; Gao et al., 2022). The result was consistent with the protection-protection factor model that high-quality peer attachment as a protective factor could enhance the beneficial effects of family cohesion and adaptability (Brook et al., 1986). Moreover, studies have shown that peer attachment, as an important social resource, could influence how individuals get along with the surrounding environment and how they cope with difficulties (Qing et al., 2018). Individuals with low peer attachment tend to exhibit negative emotions and behaviors in peer relationships. Due to the low levels of acceptance and lack of social support from peers, they feel that they are not worthy of being loved and tend to have negative representations of themselves and others. Thus, they doubt and distrust both themselves and the external environment. Therefore, individuals with low peer attachment are more likely to develop negative automatic thoughts and also exhibit a non-adaptive coping style when perceiving stressors or the lure of addictive cues such as mobile phones, which increases their risk of indulging in mobile phones. Conversely, high peer attachment could enhance individuals’ positive cognition and problem-solving skills, which reduces the risk of developing automatic thoughts and indulging in mobile phones (Yang et al., 2016). The protective role of peer attachment in our study was also consistent with the social main effect model indicating that social support has a generally beneficial effect, regardless of the current levels of social support. As long as social support is increased, it will inevitably lead to the improvement of individuals’ cognition and social adaptability (Higgins and Kruglanski, 1996). Specifically, peer attachment enhanced the protective role of family cohesion and adaptability as a significant social resource. Individuals who live in high family cohesion and adaptability would possess social resources to develop a positive cognitive model and be less indulged in mobile phones, and this effect is greater especially when they have a high-quality peer attachment relationship. Therefore, individuals with high peer attachment could enhance the positive effects of family cohesion and adaptability on individuals’ thinking styles and problem-solving skills, which, in turn, could help them alleviate automatic thoughts and reduce the occurrence of problematic behaviors.

## 5. Limitations and implications

Several limitations should be considered when interpreting these results. First, the social desirability of the self-report method used in this study may lead to imprecise data and possible common method biases. In the later stage, multiple methods such as parent or peer reports and diary methods should be considered to obtain more comprehensive data to make the results more accurate. Second, our study mainly focused on the effects of environmental (family, peers) and cognitive factors (automatic thoughts) on university students’ mobile phone addiction. In addition to these factors, emotions were found to be closely related to individuals’ family and behavioral problems (Pan and Yang, 2021). Future research should extend this model to examine the multiple mediating effects of cognitive and emotional factors.

Despite these limitations, this study expanded our knowledge of family functioning theory, cognitive emotion theory, and the protective-protective factor model which enriched our understanding of the relationship among family cohesion and adaptability, automatic thoughts, peer attachment, and mobile phone addiction. Specifically, based on these theories mentioned above, this study constructed a moderated mediation model in which family cohesion and adaptability was associated with mobile phone addiction, automatic thoughts were considered as a mediator, and peer attachment was treated as a mediator. The results not only investigated the underlying mechanism of how family cohesion and adaptability reduced individuals from indulging in mobile phones but also illustrated for whom this mediation mechanism was more prominent. The results were helpful for us to understand that family cohesion and adaptability and peer attachment can contribute to reducing individuals’ negative automatic thoughts and mobile phone addiction.

In addition, our findings also have several applications. First, faced with the detrimental effect of mobile phone addiction, educators and parents should try their best to create a positive family environment. It can promote children’s positive cognitive modes, enhance their ability to resist the lure of mobile phones, and then reduce their possibility of indulging in mobile phones. Creating a harmonious family atmosphere is better than preventing children from using mobile phones or eliminating the triggers for mobile phone addiction. Only in this way can children improve their ability to resist the lure of digital devices and better accommodate the living environment full of digital devices in the era of mobile Internet. Specifically, we also should pay more attention to individuals with low family cohesion and adaptability, formulate preventive intervention plans to encourage their parents to understand the prominent role of family function on their children’s development, and guide them to improve the family environment, so as to promote their children’s psychological adaptation. Besides, the findings of the present study reminded educators and guardians to promote individuals’ adaptive cognition through school education, community publicity, and media, so as to reduce the occurrence of problematic behaviors. Moreover, young people should be encouraged to communicate with their peers and establish close relationships or secure peer attachment to gain more peer acceptance and social support and enhance their ability to cope with setbacks. High-quality peer attachment can not only enhance the protective impact of positive family environments on individuals’ cognitive patterns but also could reduce their problematic behaviors, such as mobile phone addiction. Last but not least, our research indicated that whether it is peer attachment or family cohesion and adaptability, as long as it increases, it will have a positive impact on individuals’ development. Specifically, for individuals with conflicting family relationships, it is particularly important to cultivate high-quality peer attachment for them to develop their positive cognitive styles and promote their behavioral adaptation. Therefore, educators should guide or help them to establish a secure peer attachment relationship and train them in the skills to obtain peer social support.

## Data availability statement

The original contributions presented in the study are publicly available. This data can be found at: https://figshare.com/articles/dataset/Data_sav/23513964.

## Ethics statement

The studies involving human participants were reviewed and approved by Central China Normal University, Ethic Committee, EC, Institutional Review Board. The patients/participants provided their written informed consent to participate in this study.

## Author contributions

S-LL, X-XC, and Q-LX contributed to conception and design of the study. X-XC and Q-LX organized the database. S-LL performed the statistical analysis. X-XC wrote the first draft of the manuscript. Q-LX wrote sections of the manuscript. X-WZ, CY, and Q-QL supervised the study. All authors contributed to the article and approved the submitted version.

## Funding

This study was supported by the National Innovation and Entrepreneurship Training Program for College Students (202210489010).

## Conflict of interest

The authors declare that the research was conducted in the absence of any commercial or financial relationships that could be construed as a potential conflict of interest.

## Publisher’s note

All claims expressed in this article are solely those of the authors and do not necessarily represent those of their affiliated organizations, or those of the publisher, the editors and the reviewers. Any product that may be evaluated in this article, or claim that may be made by its manufacturer, is not guaranteed or endorsed by the publisher.
